# Supplementary material for: Bypassing spike sorting: Density-based decoding using spike localization from dense multielectrode probes
Source: bioRxiv. 2023 Sep 22:2023.09.21.558869. Preprint. [Version 1] doi: 10.1101/2023.09.21.558869 (PMC10542538; doi:10.1101/2023.09.21.558869)
Supplement: Supplement 1 [file NIHPP2023.09.21.558869v1-supplement-1.pdf]

bioRxiv preprint doi: https://doi.org/10.1101/2023.09.21.558869; this version posted September 22, 2023. The copyright holder for this preprint (which was not certified by peer review) is the author/funder, who has granted bioRxiv a license to display the preprint in perpetuity. It is made available under aCC-BY 4.0 International license.

Supplementary Material

1 Decoding using automatic differentiation variational inference (ADVI)

In the method section of our paper, we describe the general encoding-decoding paradigm. In this section of the supplementary material, we delve into a specific case that focuses on decoding continuous behaviors, $y_k \in \mathbb{R}^T$, that exhibit temporal variations. We introduce the use of ADVI, which allows us to model the relationship between firing rates of MoG components and behavior correlates using a generalized linear model (GLM).

	Notation	Definition
	s_{itk}	spike feature i at time bin t in trial k
	y_k	behavior in trial k
	z_{itk}	MoG assignment of spike feature i at time bin t in trial k
	$\eta_c = (\mu_c, \Sigma_c)$	mean and covariance matrix of MoG component c
	π_{tk}	MoG mixing proportion at time bin t in trial k
	n_{tk}	number of spikes collected at time bin t in trial k
	C	number of MoG components
	T	number of time bins within each trial
	K	number of trials in a session
	λ_{ctk}	behavior-dependent firing rate of component c at time bin t in trial k
	b_c	"baseline firing rate" of component c
ADVI	β_{ct}	"behavior-modulated firing rate" of component c at time bin t
	$\eta_{b_c} = (\mu_{b_c}, \sigma^2_{b_c})$	mean and variance of the variational posterior distribution for b_c
	$\eta_{\beta_{ct}} = (\mu_{\beta_{ct}}, \sigma^2_{\beta_{ct}})$	mean and variance of the variational posterior distribution for β_{ct}
	$\eta_{y_{tk}} = (\mu_{y_{tk}}, \sigma^2_{y_{tk}})$	mean and variance of the variational posterior distribution for y_{tk}
	$\lambda_{ct1}, \lambda_{ct0}$	firing rate of component c at time bin t that switches between two states
	ϕ	variational paramter that represents the probability of $y_k = 1$
CAVI	ρ_{ictk}	unnormalized posterior probability of assigning s_{itk} to component c
	r_{ictk}	normalized posterior probability of assigning s_{itk} to component c
	y^*_{k1}, y^*_{k0}	unnormalized posterior probability of $y_k = 1$ and $y_k = 0$
	ν_k	normalized posterior probability of $y_k = 1$

Table 2: Table of notation.

1.1 ADVI-based encoder

Building upon the general model specification outlined in Equations 1-3, we can describe the generative model of the encoder as follows:

$$\lambda_{ctk} = \exp(b_c + \beta_{ct} \cdot y_{tk}),\ b_c \sim \mathcal{N}(b_c; 0, 1),\ \beta_{ct} \sim \mathcal{N}(\beta_{ct}; 0, 1), \tag{6}$$

$$z_{itk} \sim \text{Categorical}(z_{itk}; \pi_{tk}),\ \pi_{tk} = \{\pi_{ctk}\}_{c=1}^{C},\ \pi_{ctk} = \frac{\lambda_{ctk}}{\sum_{c'} \lambda_{c'tk}}, \tag{7}$$

$$s_{itk} \sim \mathcal{N}(s_{itk}; \eta_{z_{itk}}),\ \eta_c = (\mu_c, \Sigma_c), \tag{8}$$

where b and β are the unknown model parameters corresponding to θ in Equation 1, and are sampled from a standard normal prior distribution. The latent spike assignment z depends on the mixing proportion π of the MoG, which is influenced by the behavior y through the firing rate λ. Intuitively, we can interpret λ_{ctk} as the "firing rate" of component c at time t in trial k, while b_c and β_c describe the "baseline firing rate" and "behavior-modulated firing rate" of component c, respectively.

bioRxiv preprint doi: https://doi.org/10.1101/2023.09.21.558869; this version posted September 22, 2023. The copyright holder for this preprint (which was not certified by peer review) is the author/funder, who has granted bioRxiv a license to display the preprint in perpetuity. It is made available under aCC-BY 4.0 International license.

To learn the latent variables z, b and β, we posit a mean-field Gaussian variational approximation

$$q(z, b, \beta) = \prod_{c,t} q(z_{ct}) q(b_c) q(\beta_{ct}) \tag{9}$$

for the posterior distribution $p(z, b, \beta \mid s, y)$. Obtaining exact updates for b and β is challenging due to the normalization term for π in Equation 7. Therefore, we employ ADVI to maximize the ELBO and utilize stochastic gradient ascent to update b and β. ADVI requires that the model be differentiable with respect to the parameters, and with a normal prior, the latent variables b and β reside in the real coordinate space and cause no issues with differentiability. The variational approximations for b and β are

$$q(b) = \prod_{c} q(b_c; \eta_{b_c}) = \prod_{c} \mathcal{N}(b_c; \eta_{b_c}),\ \eta_{b_c} = (\mu_{b_c}, \sigma^2_{b_c}), \tag{10}$$

$$q(\beta) = \prod_{c,t} q(\beta_{ct}; \eta_{\beta_{ct}}) = \prod_{c,t} \mathcal{N}(\beta_{ct}; \eta_{\beta_{ct}}),\ \eta_{\beta_{ct}} = (\mu_{\beta_{ct}}, \sigma^2_{\beta_{ct}}). \tag{11}$$

A drawback of the parameterization in Equations 6-8 is that the spike assignment variables z are discrete and not compatible with ADVI. An alternative, equivalent parameterization that addresses these problems is to marginalize over z. The marginalized model is

$$\lambda_{ctk} = \exp(b_c + \beta_{ct} \cdot y_{tk}),\ b_c \sim \mathcal{N}(b_c; 0, 1),\ \beta_{ct} \sim \mathcal{N}(\beta_{ct}; 0, 1), \tag{12}$$

$$\pi_{ctk} = \frac{\lambda_{ctk}}{\sum_{c'} \lambda_{c'tk}},\ \pi_{tk} = \{\pi_{ctk}\}_{c=1}^{C}, \tag{13}$$

$$s_{itk} = \sum_{c=1}^{C} \pi_{ctk} \mathcal{N}(s_{itk}; \eta_c),\ \eta_c = (\mu_c, \Sigma_c). \tag{14}$$

Under this parameterization, the ELBO for the encoder is

$$\mathcal{L}^{\text{ADVI}}_{\text{enc}} := \mathbb{E}_{q(b,\beta)}[\log p(s, b, \beta \mid y)] - \mathbb{E}_{q(b,\beta)}[\log q(b, \beta)] \tag{15}$$

$$= \mathbb{E}_{q(b,\beta)}\Big[\sum_{k,t,c,i} \log \mathcal{N}(s_{itk}; \eta_c) + \log \pi_{ctk} + \log \mathcal{N}(b_c; 0, 1) + \log \mathcal{N}(\beta_{ct}; 0, 1)\Big] \tag{16}$$

$$- \mathbb{E}_{q(b,\beta)}\Big[\sum_{c,t} \log \mathcal{N}(b_c; \eta_{b_c}) + \log \mathcal{N}(\beta_{ct}; \eta_{\beta_{ct}})\Big].$$

1.2 ADVI-based decoder

The decoder adopts the same generative model as the encoder described in Equations 6-8 with two exceptions: 1) The latent variable y_{tk} is assumed to have a standard normal prior, i.e., $y_{tk} \sim \mathcal{N}(y_{tk}; 0, 1)$, assuming independence at each time step t. Alternatively, a Gaussian process prior can be chosen to capture temporal correlations between time steps. 2) The parameters b and β are learned from the ADVI-based encoder and kept fixed in the decoder.

The mean-field Gaussian variational approximation for the posterior distribution $p(z, y \mid s)$ is

$$q(z, y) = \prod_{c,t} q(z_{ct}) q(y_t), \tag{17}$$

where

$$q(y) = \prod_{k,t} q(y_{tk}; \eta_{y_{tk}}) = \prod_{k,t} \mathcal{N}(y_{tk}; \eta_{y_{tk}}),\ \eta_{y_{tk}} = (\mu_{y_{tk}}, \sigma^2_{y_{tk}}). \tag{18}$$

To enable the use of ADVI, we can marginalize out the discrete latent variable z, thereby transforming the MoG model into a differentiable form. Under the marginalized MoG parametrization in Equations 12-14,

bioRxiv preprint doi: https://doi.org/10.1101/2023.09.21.558869; this version posted September 22, 2023. The copyright holder for this preprint (which was not certified by peer review) is the author/funder, who has granted bioRxiv a license to display the preprint in perpetuity. It is made available under aCC-BY 4.0 International license.

the ELBO of the decoder is

$$\mathcal{L}_{\text{dec}}^{\text{ADVI}} := \mathbb{E}_{q(y)}[\log p(s, y)] - \mathbb{E}_{q(y)}[\log q(y)] \tag{19}$$

$$= \mathbb{E}_{q(y)}\Big[\sum_{k,t,c,i} \log \mathcal{N}(s_{itk}; \eta_c) + \log \pi_{ctk} + \log \mathcal{N}(y_{tk}; 0, 1)\Big] \tag{20}$$

$$- \mathbb{E}_{q(y)}\Big[\sum_{k,t} \log \mathcal{N}(y_{tk}; \eta_{y_{tk}})\Big].$$

2 Decoding using coordinate ascent variational inference (CAVI)

We present a specific scenario for decoding binary variables, $y_k \in \{0, 1\}$, where we derive exact updates for the variational variables using CAVI.

2.1 CAVI-based encoder

Extending the general model described in Equations 1-3, the generative model of the encoder can be defined as follows:

$$\pi_{ctk} = \left(\frac{\lambda_{ct1}}{\sum_{c'} \lambda_{c't1}}\right)^{y_k} \left(\frac{\lambda_{ct0}}{\sum_{c'} \lambda_{c't0}}\right)^{1-y_k}, \tag{21}$$

$$z_{itk} \sim \text{Categorical}(z_{itk}; \pi_{tk}),\ \pi_{tk} = \{\pi_{ctk}\}_{c=1}^{C}, \tag{22}$$

$$s_{itk} \sim \mathcal{N}(s_{itk}; \eta_{z_{itk}}),\ \eta_c = (\mu_c, \Sigma_c), \tag{23}$$

where z and λ are the latent variables that we aim to learn. The behavior-dependent firing rates of each component c at time t vary based on the binary variable y, such that the components switch between two behavioral states characterized by firing rates λ_{ct1} and λ_{ct0}.

The log-likelihood of the encoder can be written as

$$\log p(s, z \mid y) = \sum_{k,t,c,i} z_{ictk}\Big\{\log \mathcal{N}(s_{itk}; \eta_c) + y_k(\log \lambda_{ct1} - \log \Lambda_{t1}) + (1 - y_k)(\log \lambda_{ct0} - \log \Lambda_{t0})\Big\}, \tag{24}$$

where $\Lambda_{t1} = \sum_{c'} \lambda_{c't1}$ and $\Lambda_{t0} = \sum_{c'} \lambda_{c't0}$. To approximate the posterior $p(z \mid s, y)$, we employ the mean-field variational approximation $q(z) = \prod_{c,t} q(z_{ct})$. The ELBO of the encoder is

$$\mathcal{L}_{\text{enc}}^{\text{CAVI}} := \mathbb{E}_{q(z)}[\log p(s, z \mid y)] - \mathbb{E}_{q(z)}[\log q(z)]. \tag{25}$$

The exact update for $q(z)$ is obtained by maximizing the ELBO with respect to $q(z)$, which leads to the following update equation:

$$q(z_{ct}) \propto \exp\{\mathbb{E}_{q_{-ct}}[\log p(z_{ct}, z_{-ct}, s \mid y)]\}, \tag{26}$$

where q_{-ct} means $\prod_{c' \neq c} \prod_{t' \neq t} q(z_{c't'})$. Then,

$$q(z_{ictk} = 1) \propto \exp\left\{\log \mathcal{N}(s_{itk}; \eta_c) + y_k \log\left(\frac{\lambda_{ct1}}{\Lambda_{t1}}\right) + (1 - y_k)\log\left(\frac{\lambda_{ct0}}{\Lambda_{t0}}\right)\right\} := \rho_{ictk} \tag{27}$$

denotes the unnormalized posterior probability of assigning spike i collected at time t in trial k to component c, while $\mathbb{E}[z_{ictk}] = \rho_{ictk} / \sum_{c'} \rho_{ic'tk} := r_{ictk}$ represents the normalized posterior probability.

After fixing $q(z)$, the term in the ELBO which depends on λ, μ and Σ can be expressed as

$$\mathscr{L} := \sum_{k,t,c,i} r_{ictk}\Big\{\log \mathcal{N}(s_{itk}; \eta_c) + y_k \log\left(\frac{\lambda_{ct1}}{\Lambda_{t1}}\right) + (1 - y_k)\log\left(\frac{\lambda_{ct0}}{\Lambda_{t0}}\right)\Big\}.$$

bioRxiv preprint doi: https://doi.org/10.1101/2023.09.21.558869; this version posted September 22, 2023. The copyright holder for this preprint (which was not certified by peer review) is the author/funder, who has granted bioRxiv a license to display the preprint in perpetuity. It is made available under aCC-BY 4.0 International license.

Algorithm 1 CAVI-based encoder

Input: $\{s_{itk}\}, \{y_k\}, i = 1 : n_{tk}, t = 1 : T, k = 1 : K$, number of components C.

```
Initialize {μ_c, Σ_c}, c = 1 : C.
while ELBO not converged do
    for all k ∈ 1 : K do
        for all t ∈ 1 : T do
            for all i ∈ 1 : n_tk do
                Set q(z_ictk = 1) ∝ ρ_ictk.                          ▷ eq. (27)
            end for
        end for
    end for

    for all c ∈ 1 : C do
        Set μ_c = μ*_c, Σ_c = Σ*_c.                                  ▷ eq. (36-39)
        for all t ∈ 1 : T do
            Set λ_ct0 = λ*_ct0, λ_ct1 = λ*_ct1.                      ▷ eq. (32-33)
        end for
    end for
    Compute the ELBO L_enc^CAVI.                                     ▷ eq. (25)
end while
Return q(z), λ, μ, Σ.
```

We derive the update for λ by setting the gradients $\nabla_{\lambda_{ct1}}\mathscr{L}$ and $\nabla_{\lambda_{ct0}}\mathscr{L}$ to 0:

$$\nabla_{\lambda_{ct1}}\mathscr{L} = \nabla_{\lambda_{ct1}} \sum_{k,i} r_{ictk} \cdot y_k(\log \lambda_{ct1} - \log \Lambda_{t1}) \tag{28}$$

$$= \frac{\sum_{k,i} r_{ictk} \cdot y_k}{\lambda_{ct1}} - \frac{\sum_{k,i} \sum_{c'=1}^{C} r_{ic'tk} \cdot y_k}{\Lambda_{t1}} = 0 \tag{29}$$

$$\implies \sum_{k,i} r_{ictk} \cdot y_k(\lambda_{ct1} + \sum_{c'\neq c} \lambda_{c't1}) = \sum_{k,i} \sum_{c'=1}^{C} r_{ic'tk} \cdot y_k \cdot \lambda_{ct1} \tag{30}$$

$$\implies \sum_{k,i} y_k(\sum_{c'\neq c} r_{ic'tk})\lambda_{ct1} = \sum_{k,i} y_k \cdot r_{ictk}(\sum_{c'\neq c} \lambda_{c't1}) \tag{31}$$

$$\implies \lambda_{ct1} = \frac{\sum_{k,i} y_k \cdot r_{ictk}(\sum_{c'\neq c} \lambda_{c't1})}{\sum_{k,i} y_k(\sum_{c'\neq c} r_{ic'tk})} := \lambda^*_{ct1}. \tag{32}$$

$$\nabla_{\lambda_{ct0}}\mathscr{L} = 0 \implies \lambda_{ct0} = \frac{\sum_{k,i}(1 - y_k) r_{ictk}(\sum_{c'\neq c} \lambda_{c't0})}{\sum_{k,i}(1 - y_k)(\sum_{c'\neq c} r_{ic'tk})} := \lambda^*_{ct0}. \tag{33}$$

Consider the gradient with respect to the η_c parameter,

$$\nabla_{\eta_c}\mathscr{L} = \sum_{k,t,i} r_{ictk} \nabla_{\eta_c} \log \mathcal{N}(s_{itk}; \eta_c) \tag{34}$$

$$= \sum_{k,t,i} r_{ictk} \nabla_{\eta_c} \frac{1}{2}\left(\log|\Sigma_c^{-1}| - \mathrm{Tr}\{\Sigma_c^{-1}(s_{itk} - \mu_c)(s_{itk} - \mu_c)^\top\}\right). \tag{35}$$

bioRxiv preprint doi: https://doi.org/10.1101/2023.09.21.558869; this version posted September 22, 2023. The copyright holder for this preprint (which was not certified by peer review) is the author/funder, who has granted bioRxiv a license to display the preprint in perpetuity. It is made available under aCC-BY 4.0 International license.

The closed-form updates for μ_c and Σ_c are

$$\nabla_{\mu_c}\mathscr{L} = \sum_{k,t,i} r_{ictk}\Sigma_c^{-1}(s_{itk} - \mu_c) = 0 \tag{36}$$

$$\implies \mu_c = \frac{1}{n_c}\sum_{k,t,i} r_{ictk}s_{itk} := \mu_c^*,\ n_c = \sum_{k,t,i} r_{ictk}. \tag{37}$$

$$\nabla_{\Sigma_c}\mathscr{L} = \frac{1}{2}\sum_{k,t,i} r_{ictk}(\Sigma_c - (s_{itk} - \mu_c)(s_{itk} - \mu_c)^\top) = 0 \tag{38}$$

$$\implies \Sigma_c = \frac{1}{n_c}\sum_{k,t,i} r_{ictk}(s_{itk} - \mu_c)(s_{itk} - \mu_c)^\top := \Sigma_c^*. \tag{39}$$

2.2 CAVI-based decoder

The CAVI-based decoder employs the same generative model as the CAVI-based encoder, with the exception that the behavior-dependent firing rates λ_{ct1} and λ_{ct0} are learned by the encoder and kept fixed, and the behavior y is treated as an unknown latent variable. We sample y from a prior distribution, $y_k \sim \text{Bernoulli}(\phi)$, where ϕ is a variational parameter that represents the probability that $y_k = 1$. The log-likelihood can be expressed as follows

$$\log p(s, z, y) = \sum_{k,t,c,i} z_{ictk}\{\log \mathcal{N}(s_{itk}; \eta_c) + y_k(\log \lambda_{ct1} - \log \Lambda_{t1}) \\ + (1 - y_k)(\log \lambda_{ct0} - \log \Lambda_{t0})\} + y_k \log \phi + (1 - y_k)\log(1 - \phi). \tag{40}$$

We use the factorization $q(z, y) = q(z)q(y) = \prod_{c,t} q(z_{ct})q(y)$ to approximate the posterior distribution $p(z, y \mid s)$. The ELBO of the decoder can be defined as

$$\mathcal{L}_{\text{dec}}^{\text{CAVI}} := \mathbb{E}_{q(z,y)}[\log p(s, z, y)] - \mathbb{E}_{q(z,y)}[\log q(z, y)]. \tag{41}$$

Algorithm 2 CAVI-based decoder

Input: $\{s_{itk}\}, \{\lambda_{ct0}, \lambda_{ct1}\}$, $i = 1 : n_{tk}$, $t = 1 : T$, $k = 1 : K$, $c = 1 : C$.
  Initialize $\{\mu_c\}, \{\Sigma_c\}$.
  while ELBO not converged **do**
    for all $k \in 1 : K$ **do**
      Set $q(y_k = 1) \propto y_{k1}^*$. ▷ eq. (44)
      for all $t \in 1 : T$ **do**
        for all $i \in 1 : n_{tk}$ **do**
          Set $q(z_{ictk} = 1) \propto \rho_{ictk}$. ▷ eq. (43)
        end for
      end for
    end for

    for all $c \in 1 : C$ **do**
      Set $\mu_c = \mu_c^*$, $\Sigma_c = \Sigma_c^*$. ▷ eq. (36-39)
    end for
    Set $\phi = \phi^*$. ▷ eq. (46)
    Compute the ELBO $\mathcal{L}_{\text{dec}}^{\text{CAVI}}$. ▷ eq. (41)
  end while
  Return $q(z, y), \phi, \mu, \Sigma$.

The exact updates for $q(z)$ and $q(y)$ that guarantee an increase in the ELBO are

$$q(z) \propto \exp\{\mathbb{E}_{q(y)}[\log p(s, z, y)]\}, \quad q(y) \propto \exp\{\mathbb{E}_{q(z)}[\log p(s, z, y)]\}, \tag{42}$$

bioRxiv preprint doi: https://doi.org/10.1101/2023.09.21.558869; this version posted September 22, 2023. The copyright holder for this preprint (which was not certified by peer review) is the author/funder, who has granted bioRxiv a license to display the preprint in perpetuity. It is made available under aCC-BY 4.0 International license.

where

$$q(z_{ictk} = 1) \propto \exp\{\log \mathcal{N}(s_{itk}; \eta_c) + \mathbb{E}[y_k] \log\left(\frac{\lambda_{ct1}}{\Lambda_{t1}}\right) + (1 - \mathbb{E}[y_k]) \log\left(\frac{\lambda_{ct0}}{\Lambda_{t0}}\right)\} := \rho_{ictk}, \tag{43}$$

and $\mathbb{E}[z_{ictk}] = \rho_{ictk} / \sum_{c'} \rho_{ic'tk} := r_{ictk}$ are the unnormalized and normalized posterior probabilities of assigning spike i collected at time t in trial k to component c, respectively. The unnormalized posterior probabilities of $y_k = 1$ and $y_k = 0$ are

$$q(y_k = 1) \propto \exp\{\sum_{t,c,i} \mathbb{E}[z_{ictk}](\log \lambda_{ct1} - \log \Lambda_{t1}) + \log \phi\} := y^*_{k1}, \tag{44}$$
$$q(y_k = 0) \propto \exp\{\sum_{t,c,i} \mathbb{E}[z_{ictk}](\log \lambda_{ct0} - \log \Lambda_{t0}) + \log(1 - \phi)\} := y^*_{k0},$$

and $\mathbb{E}[y_k] = y^*_{k1} / (y^*_{k1} + y^*_{k0}) := \nu_k$ represents the normalized posterior probability of $y_k = 1$.

After fixing $q(z)$ and $q(y)$, the term in the ELBO which depends on ϕ, μ and Σ can be written as

$$\begin{aligned} \mathcal{L}' := \sum_{k,t,c,i} r_{ictk}\{\log \mathcal{N}(s_{itk}; \eta_c) + \nu_k(\log \lambda_{ct1} - \log \Lambda_{t1}) \\ + (1 - \nu_k)(\log \lambda_{ct0} - \log \Lambda_{t0})\} + \nu_k \log \phi + (1 - \nu_k) \log(1 - \phi). \end{aligned} \tag{45}$$

Considering the gradient of the ELBO with respect to the ϕ parameter, we obtain its update:

$$\nabla_\phi \mathcal{L}' = 0 \implies \phi^* = \frac{1}{K} \sum_{k=1}^{K} \nu_k. \tag{46}$$

The updates for η_c are computed in a similar manner as described in Equations 36-39.

3 MoG initialization

We employ the following procedure to intialize the MoG model used in both the ADVI-based and CAVI-based algorithms: 1) According to Figure 1 (a), the spike feature distribution is highly multimodal. To determine the appropriate number of modes, we utilize *isosplit* (Magland et al. 2015) to cluster the spike features. This step helps in splitting the set of spike features into distinct clusters. 2) For each identified cluster, we compute the mean and variance of the spike features belonging to that cluster, which serve as the parameters for the corresponding Gaussian component. This automatic selection of the number of MoG components and the initialization of means and covariance matrices facilitate the initialization of the ADVI-based and CAVI-based algorithms.

4 Data preprocessing

We provide a brief overview of our data preprocessing pipeline, which involves the following steps.

Destriping. During the data collection process, we encounter line noise due to voltage leakage on the probe. This translates into large "stripes" of noise spanning the whole probe. To mitigate the impact of these noise artifacts, we apply a destriping procedure (Chapuis et al. 2022).

Subtraction-based spike detection and denoising. We employ the iterative subtraction-based procedure for spike detection and collision-correction described in Boussard et al. 2023.

Spike localization. We employ the method of Boussard et al. 2021 to estimate the location of each denoised spike.

bioRxiv preprint doi: https://doi.org/10.1101/2023.09.21.558869; this version posted September 22, 2023. The copyright holder for this preprint (which was not certified by peer review) is the author/funder, who has granted bioRxiv a license to display the preprint in perpetuity. It is made available under aCC-BY 4.0 International license.

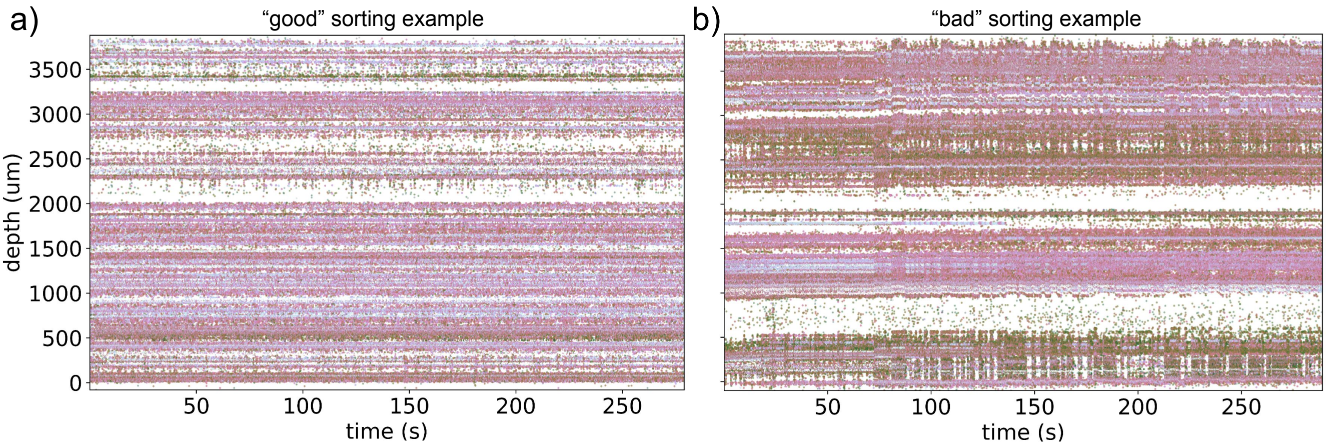

Figure 6: **Motion drift in "good" and "bad" sorting recordings.** (a) The motion-registered spike raster plot of a "good" sorting example that is less affected by drift. (b) The spike raster plot of a "bad" sorting example, which is still affected by drift even after registration.

Drift registration. Probe motion (or drift) in the electrophysiology data poses a challenge for downstream analyses. Decentralized registration (Windolf et al. 2022) is applied to track and correct for motion drift in the high-density probe recordings.

5 Behavior decoder

To decode binary behaviors, such as the mouse's left or right choices, we utilize $\mathcal{L}_2$-penalized logistic regression. For decoding dynamic behaviors, such as wheel speed, we employ a sliding-window algorithm to aggregate the entries of the weight matrix, W_{kct}, over time. Within the time window $[t-\delta, t+\delta]$, where δ is the window size, we stack 2δ weight matrix entries, $\{W_{kct}\}_{c=1}^{C}$, for time point t in trial k. This aggregated weight matrix is then used as input for ridge regression to predict the behavior y_{tk} at time t. The window size δ and the regularization strength are model hyper-parameters, set through cross-validation to achieve the optimal decoding performance.

bioRxiv preprint doi: https://doi.org/10.1101/2023.09.21.558869; this version posted September 22, 2023. The copyright holder for this preprint (which was not certified by peer review) is the author/funder, who has granted bioRxiv a license to display the preprint in perpetuity. It is made available under aCC-BY 4.0 International license.

6 Model interpretation

In this section, we provide visualizations to gain insights into the effectiveness of our proposed decoder. We quantify the posterior entropy of each spike assignment in Figure 7 (a). Spike assignments with higher entropy correspond to a spread of posterior probabilities among multiple MoG components. In contrast, traditional spike sorting or thresholding methods result in deterministic spike assignments, leading to lower entropy and empirically reduced decoding performance. In Figure 7 (b), we compare the trial-averaged weight matrices (W) used for decoding between spike-sorted, spike-thresholded, and our proposed decoders.

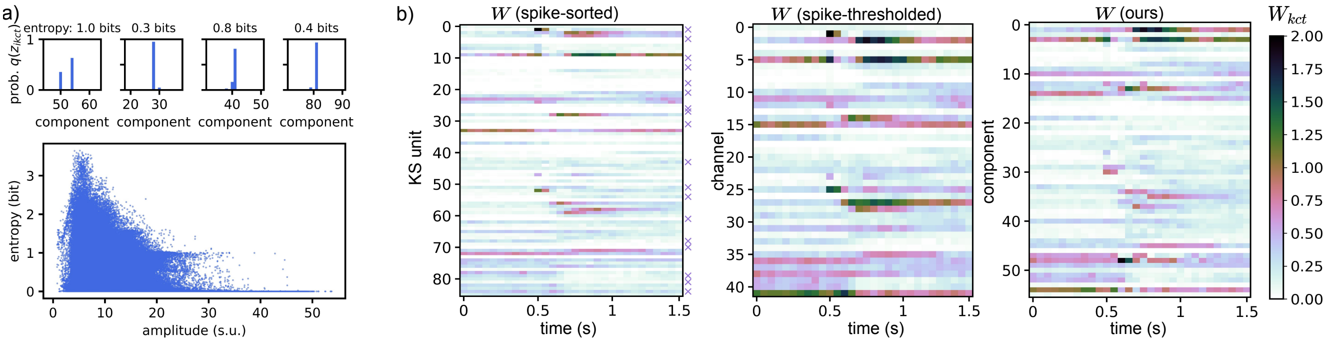

Figure 7: **Model interpretation.** (a) The posterior entropy of the spike assignment is high, when the posterior probability of spike assignment, $q(z_{ikct})$, is spread out among several MoG components instead of being concentrated at a single component. The scatter plot shows that low-amplitude spikes that are difficult to assign have higher posterior entropy than high-amplitude spikes. (b) Visualizations of the averaged weight matrices W's across trials in an example IBL session. For "W (spike-sorted and spike-thresholded)", the W matrix has one-hot rows and each entry W_{kct} is the spike count that belongs to KS unit (channel) c and time bin t in trial k. The purple crosses indicate the "good" KS units. For "W (ours)", W_{kct} is the sum of posterior probabilities of spike assignments, and the MoG mixing proportion π depends on the behavior y and changes over time. The arrangement of KS units (channels or MoG components) on the heat maps is based on the depth of the NP probe, ensuring comparability across the displayed W matrices.

7 Decoding across brain regions

In addition to the previously mentioned five brain regions (PO, LP, DG, CA1, VISa) depicted in Figure 3, we expanded our analysis to include two additional brain regions situated in the cerebellum: the arbor vitae (ARB) and the ansiform cruciform lobule (ANCR). We specifically include the cerebellum in our analysis due to the frequent occurrence of spike sorting issues in this area. In Figure 8 and 9, we present a comparison between our decoder and the spike-sorted decoder that utilizes all KS units across all the brain regions studied. According to Figure 8, our method consistently outperforms the decoder that relies on all KS units across all brain regions and the majority of IBL sessions. Furthermore, Figure 9 specifically demonstrates that our method consistently achieves superior decoding performance in the recorded regions of the cerebellum, where spike sorting quality issues are commonly encountered. This highlights the robustness and reliability of our method, particularly in challenging recording conditions.

bioRxiv preprint doi: https://doi.org/10.1101/2023.09.21.558869; this version posted September 22, 2023. The copyright holder for this preprint (which was not certified by peer review) is the author/funder, who has granted bioRxiv a license to display the preprint in perpetuity. It is made available under aCC-BY 4.0 International license.

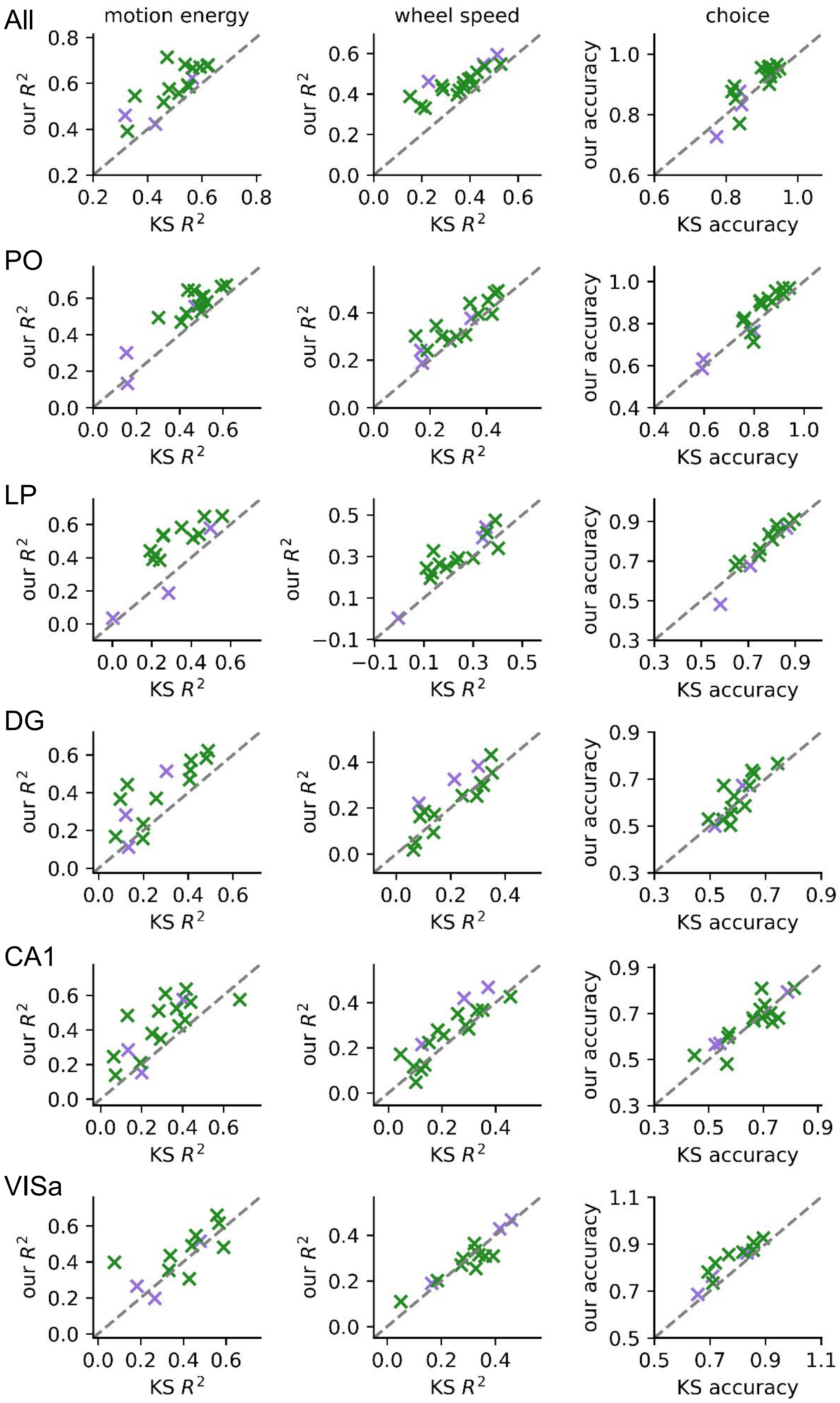

Figure 8: **Decoding comparisons across brain regions in the thalamus, hippocampus and visual cortex.** We compare our decoder to the spike-sorted decoder using all KS units across various brain regions and behavioral tasks. Each point in the scatter plot represents one session from the 20 IBL session previously described in the experiments section. Sessions with "good" sorting quality are depicted in green, while sessions with "bad" sorting quality are shown in purple. The majority of the sessions lie above the gray diagonal line, indicating that our method consistently outperforms the decoder relying on all KS units.

bioRxiv preprint doi: https://doi.org/10.1101/2023.09.21.558869; this version posted September 22, 2023. The copyright holder for this preprint (which was not certified by peer review) is the author/funder, who has granted bioRxiv a license to display the preprint in perpetuity. It is made available under aCC-BY 4.0 International license.

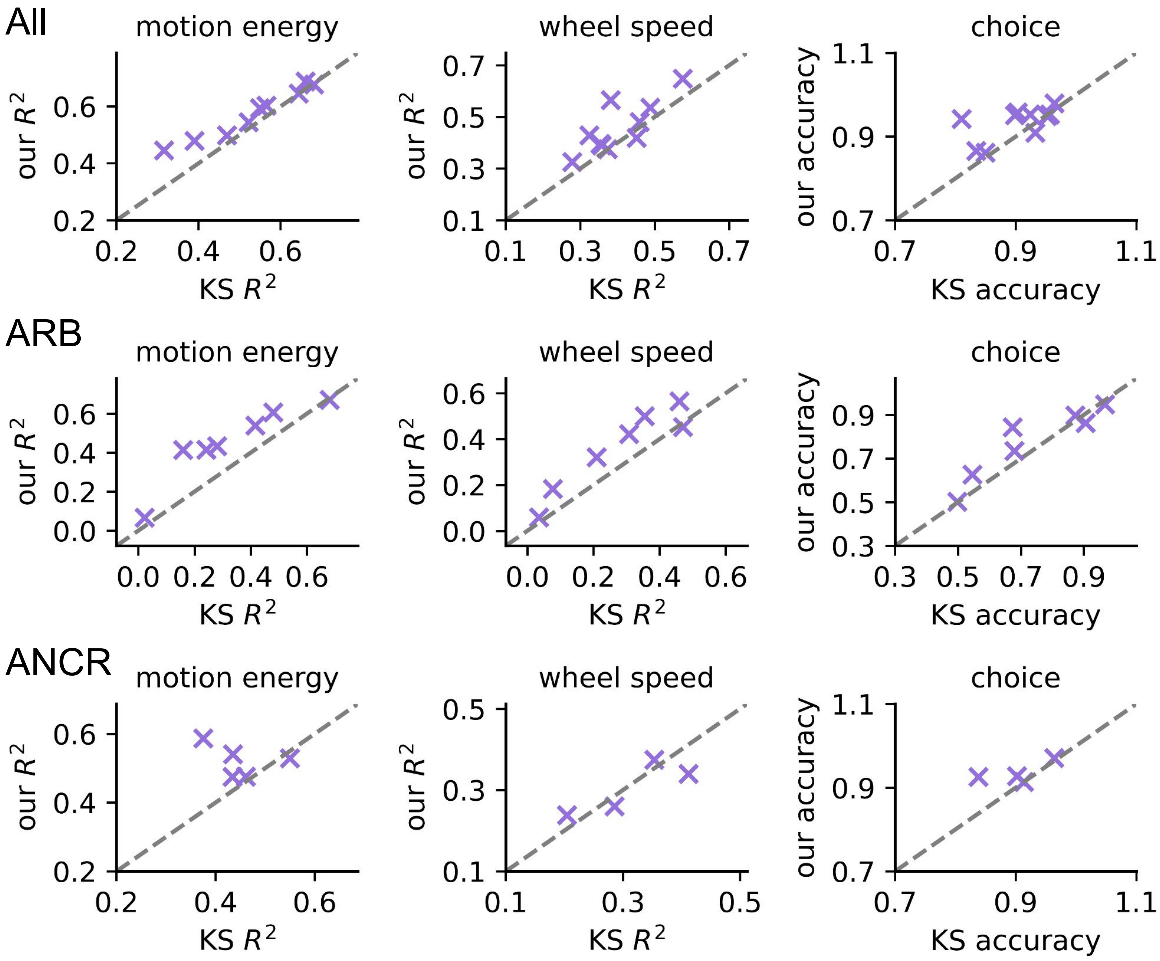

Figure 9: **Decoding comparisons across brain regions in the cerebellum.** We evaluate our decoder against the spike-sorted decoder utilizing all KS units across various brain regions in the cerebellum. The scatter plot visualizes the results for each IBL session in the study, with all sessions depicted in purple indicating "bad" sorting quality. Note that the probes used in the cerebellum sessions were not uniformly implanted in the same set of brain regions. As a result, the number of available sessions for decoding in certain cerebellum regions is limited. Notably, the majority of sessions are positioned above the gray diagonal line, indicating that our method consistently achieves better decoding performance compared to the decoder relying on all KS units. This outcome highlights the robustness of our method, particularly in the context of the cerebellum where spike sorting quality issues are prevalent.

bioRxiv preprint doi: https://doi.org/10.1101/2023.09.21.558869; this version posted September 22, 2023. The copyright holder for this preprint (which was not certified by peer review) is the author/funder, who has granted bioRxiv a license to display the preprint in perpetuity. It is made available under aCC-BY 4.0 International license.

8 Ablation study

We conduct an ablation study to investigate the importance of various components in our decoding paradigm. Specifically, we examine how the integration of dynamic (behavior-dependent) mixing proportions in the MoG and higher-dimensional spike features influence decoding performance. Additionally, we analyze how the inclusion criteria for spike waveforms can affect decoding outcomes.

Effects of dynamic MoG mixing proportion. Table 3 presents a comparison between the ordinary MoG with a fixed mixing proportion (referred to as "fixed π") and our proposed model with a dynamic mixing proportion (referred to as "dynamic π"). The results indicate that this approach leads to improved decoding performance compared to using a fixed mixing proportion in the MoG model.

	Motion energy (R^2)		Wheel speed (R^2)		Choice (accuracy)	
	Fixed π	Dynamic π	Fixed π	Dynamic π	Fixed π	Dynamic π
All	0.664 (± 0.034)	**0.742** (± 0.028)	0.470 (± 0.062)	**0.564** (± 0.045)	0.948 (± 0.038)	**0.957** (± 0.036)
PO	0.365 (± 0.031)	**0.488** (± 0.046)	0.520 (± 0.019)	**0.670** (± 0.015)	0.844 (± 0.016)	**0.861** (± 0.035)
LP	0.145 (± 0.015)	**0.464** (± 0.054)	0.114 (± 0.032)	**0.342** (± 0.027)	0.917 (± 0.026)	**0.931** (± 0.022)
DG	0.280 (± 0.033)	**0.492** (± 0.042)	0.221 (± 0.040)	**0.381** (± 0.035)	0.669 (± 0.084)	**0.722** (± 0.050)
CA1	0.407 (± 0.021)	**0.538** (± 0.038)	0.308 (± 0.051)	**0.428** (± 0.030)	0.621 (± 0.062)	**0.626** (± 0.064)
VISa	0.488 (± 0.046)	**0.490** (± 0.047)	0.318 (± 0.056)	**0.364** (± 0.074)	0.857 (± 0.065)	**0.874** (± 0.066)

Table 3: Effects of dynamic mixing proportion of the MoG on decoding performance.

Effects of higher-dimensional spike features. In Table 4, we provide a comparison of decoding performance using two different sets of spike features. The first set includes spike location along the width and depth dimensions of the probe (denoted as x and z) as well as the maximum peak-to-peak amplitude of the spike (denoted as a). The second set includes the first and second principal components (PCs) of the spike waveforms (denoted as u_1 and u_2) in addition to x, z and a. The spike features are visually represented using scatter plots in Figure 10. We report the mean and standard deviation of the decoding accuracy (R^2) obtained from a 5-fold CV on a single session. Table 4 provides insights regarding the inclusion of additional waveform PC features for decoding. The findings suggest that the incorporation of these additional PC features does not contribute to significant improvements in decoding performance.

	Motion energy (R^2)		Wheel speed (R^2)		Choice (accuracy)	
	(x, z, a)	(x, z, a, u_1, u_2)	(x, z, a)	(x, z, a, u_1, u_2)	(x, z, a)	(x, z, a, u_1, u_2)
All	**0.531** (± 0.026)	0.529 (± 0.026)	**0.484** (± 0.042)	0.478 (± 0.045)	0.917 (± 0.019)	0.917 (± 0.019)
PO	**0.462** (± 0.038)	0.457 (± 0.039)	**0.469** (± 0.061)	0.464 (± 0.051)	0.853 (± 0.019)	0.853 (± 0.025)
LP	**0.490** (± 0.026)	0.489 (± 0.029)	**0.479** (± 0.035)	0.473 (± 0.013)	**0.864** (± 0.022)	0.849 (± 0.036)
DG	**0.335** (± 0.028)	0.321 (± 0.037)	**0.273** (± 0.030)	0.264 (± 0.025)	0.675 (± 0.038)	**0.679** (± 0.049)
CA1	**0.449** (± 0.027)	0.440 (± 0.046)	**0.329** (± 0.044)	0.328 (± 0.040)	0.755 (± 0.045)	**0.758** (± 0.053)
VISa	**0.270** (± 0.021)	0.237 (± 0.017)	**0.225** (± 0.024)	0.206 (± 0.013)	0.725 (± 0.048)	**0.732** (± 0.054)

Table 4: Effects of incorporating higher-dimensional spike features on decoding performance.

bioRxiv preprint doi: https://doi.org/10.1101/2023.09.21.558869; this version posted September 22, 2023. The copyright holder for this preprint (which was not certified by peer review) is the author/funder, who has granted bioRxiv a license to display the preprint in perpetuity. It is made available under aCC-BY 4.0 International license.

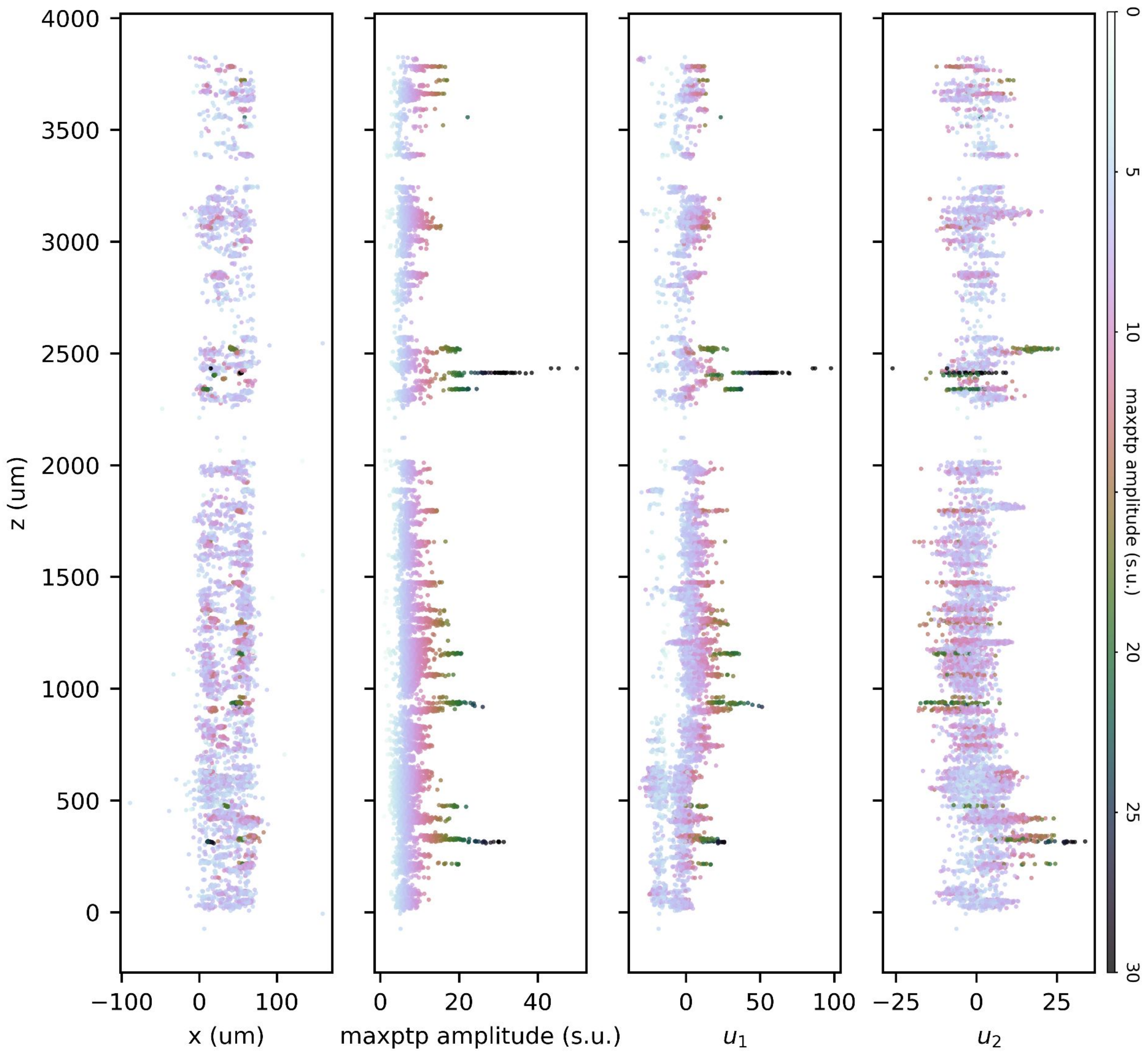

Figure 10: **Visualizations of spike features employed for decoding.** Spike localization features, (x, z), the locations of spikes along the width and depth of the NP1 probe, and waveform features, a, the maximum peak-to-peak (max ptp) amplitudes of spikes. Amplitude is measured in standard units (s.u.). u_1 and u_2 denote the first and second principal components (PCs) of the spike waveforms.

bioRxiv preprint doi: https://doi.org/10.1101/2023.09.21.558869; this version posted September 22, 2023. The copyright holder for this preprint (which was not certified by peer review) is the author/funder, who has granted bioRxiv a license to display the preprint in perpetuity. It is made available under aCC-BY 4.0 International license.

Effects of inclusion criteria for spike waveforms. To investigate whether the density-based decoder is performing better by using additional spikes that a spike sorter would miss, we conducted an experiment using different inclusion criteria for spike waveforms. We fitted our model using only spikes detected by Kilosort 2.5, and compared its performance to decoders using spike-sorted outputs and our subtraction-based spike detection on choice and motion energy decoding. The results are summarized in Table 5. As shown in the table, our decoder can achieve comparable or better decoding performance than the spike-sorted decoder when modeling the same spikes. This suggests that the gain in decoding performance can be attributed to the density-based approach, instead of the spike inclusion criteria.

	Choice (accuracy)	Motion energy (R^2)
Density-based (subtraction spikes)	0.876 (± 0.068)	**0.589** (± 0.111)
Density-based (KS spikes)	0.876 (± 0.079)	0.579 (± 0.121)
Sorted (KS spikes)	**0.887** (± 0.078)	0.503 (± 0.117)

Table 5: Effects of inclusion criteria for spike waveforms on decoding performance.

9 Comparison to a state-of-the-art clusterless decoder

In this section, we outline the specific experimental setup for evaluating our density-based decoder in comparison with the clusterless point process decoder (Denovellis et al. 2021) on both multiple tetrodes and high-density (HD) probes data.

Application to tetrodes. We utilized the code provided in the GitHub repository* of the clusterless point process decoder (Denovellis et al. 2021) to generate simulated neural and behavioral data. This synthetic dataset was designed to mimic recordings from 5 tetrodes, with each tetrode containing 4 channels. Spike amplitudes from each channel of these multiple tetrodes were selected as the spike features for both decoding methods. The objective of the decoding was to estimate the animal's position from these simulated spike features. As the original simulated position was too straightforward to decode, we intentionally distorted it by blending it with real position data sourced from the GitHub repository†. Moreover, we introduced random Gaussian noise to the simulated position for added complexity.

Application to HD probes. We evaluated both decoders on decoding wheel speed and motion energy from spike features extracted from NP1 probes across three IBL datasets. To preprocess the data, we followed the pipeline outlined above, extracting spike localization features and maximum peak-to-peak amplitudes as common features for both decoders.

We only focus on continuous behaviors for decoding since the clusterless point process decoder is designed exclusively for continuous behaviors. Notably, due to its continuous time nature, the clusterless point process decoder directly decoded behaviors without time binning. In contrast, our density-based model required time binning of both behavioral and spike feature data into equal-sized time intervals. Consequently, we used the time-binned behaviors for decoding with the density-based approach. We employed 5-fold cross-validation to assess the decoding performance of both decoders. We used a random walk as the state transition for the clusterless point process decoder, and used the estimated variance from the animal's behavior to set the variance of the random walk. The clusterless point process decoder uses a grid-based approximation of the inferred behavior, discretizing the behavior space into place bins; see Section "Decoding" in Denovellis et al. 2021. We determine place bin size based on the square root of the observed behavior variance. Denovellis et al. 2021 use kernel density estimation (KDE) to estimate the distributions of both the behavior variable and the spike features used for decoding. We set the KDE bandwidth that determines the amount of smoothing done for spike features to be 1.0, and the bandwidth for behavior to be the square root of the observed behavior variance; see Section "Encoding - clusterless" in Denovellis et al. 2021.

*https://github.com/Eden-Kramer-Lab/replay_trajectory_classification
†https://github.com/nelpy/example-analyses/blob/master/LinearTrackDemo.ipynb

bioRxiv preprint doi: https://doi.org/10.1101/2023.09.21.558869; this version posted September 22, 2023. The copyright holder for this preprint (which was not certified by peer review) is the author/funder, who has granted bioRxiv a license to display the preprint in perpetuity. It is made available under aCC-BY 4.0 International license.

10 Simulation for model validation

We conducted simulations to illustrate the principles of our method. The simulation aimed to show that our encoding model can learn the relationship between spike features and behaviors. We performed two tasks, decoding a binary variable, y_k, simulated from a Bernoulli distribution, and decoding a continuous variable, y_k, simulated from a Gaussian process. To mimic the data-generating process, we selected Gaussian components with "templates" extracted from a real dataset. The encoding model parameters, b and β, were also taken from learned parameters in the same dataset. Given b, β and y_k, we simulated the "firing rates" λ for each Gaussian component in the mixture, as described in the Method section of our paper. Next, we generated spike features based on these simulated "firing rates," and applied the encoding model to infer the behavior-dependent λ. Figure 11 displays the learned λ for each component c, time t, and trial k. The learned "firing rates" closely resembled the simulated ones, indicating the model's ability to recover the primary associations between spike features and behaviors. With such associations, the decoding model can decode behaviors.

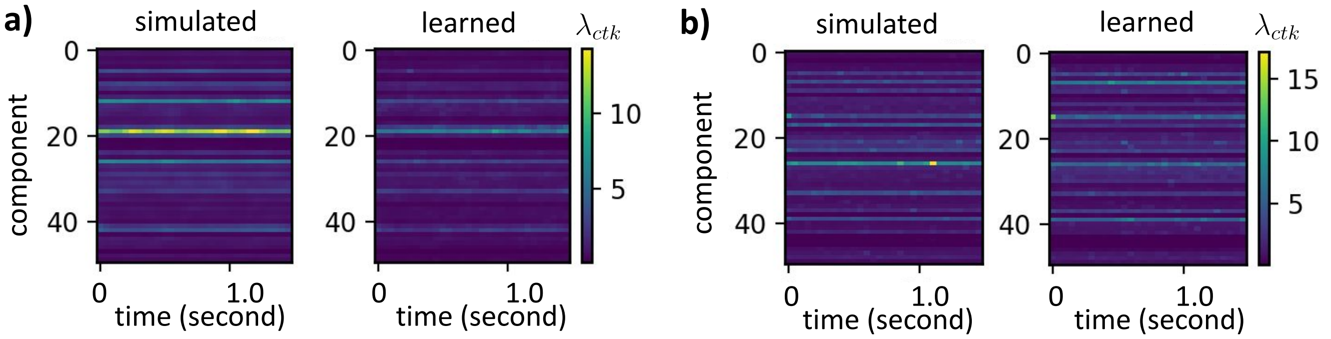

Figure 11: **Our encoding model recovers the relationship between the simulated spiking activity and the simulated behavior correlate.** Panel (a) shows a comparison of the simulated firing rates conditioned on the binary behavior variable with the learned firing rates by our encoding model. In Panel (b), we compare the simulated firing rates conditioned on the continuous behavior variable with the learned firing rates from our encoding model.

bioRxiv preprint doi: https://doi.org/10.1101/2023.09.21.558869; this version posted September 22, 2023. The copyright holder for this preprint (which was not certified by peer review) is the author/funder, who has granted bioRxiv a license to display the preprint in perpetuity. It is made available under aCC-BY 4.0 International license.

11 Relationship to spike sorting

We conducted experiments to investigate the biological interpretation of our MoG units and the correspondence between single cells identified by KS and our MoG units. The agreement matrix between "hard" KS spike assignments and "soft" MoG assignments is shown in Figure 12. We calculated the conditional probability of spikes belonging to each MoG component, given that these spikes belong to the corresponding KS unit. Notably, KS units with large amplitudes are less likely to be split into multiple Gaussian components. This shows a reasonable correspondence between the Gaussian components and the spike-sorted units.

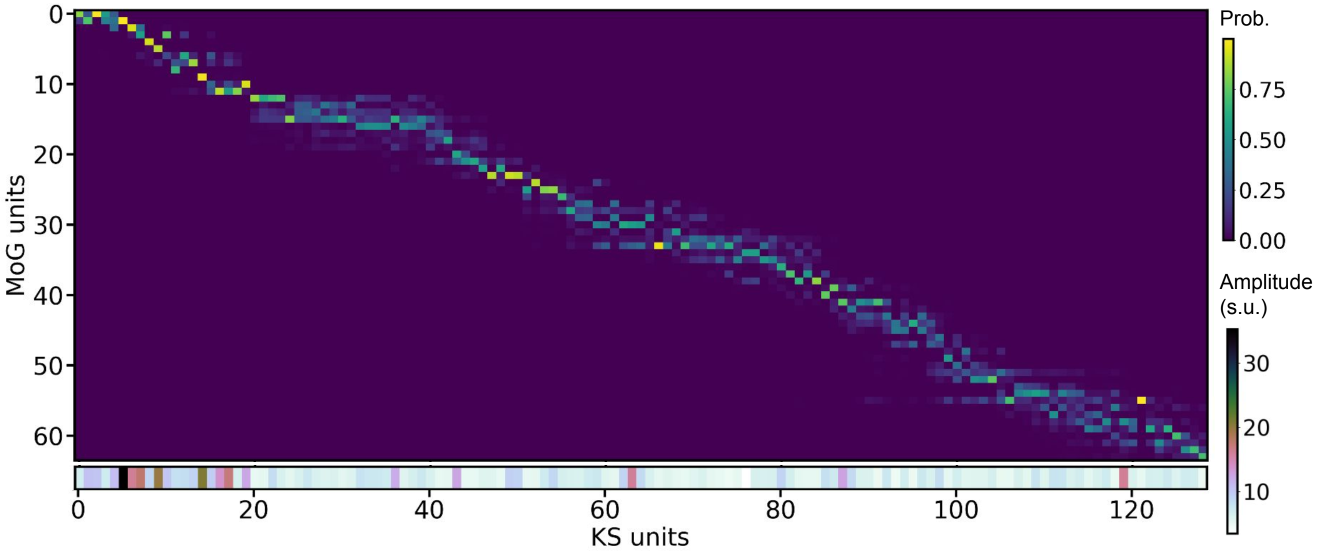

Figure 12: **Correspondence between Kilosort and MoG spike assignment**. Units are ordered by their depth on the Neuropixel probe. The color bar shows the conditional probability of spikes belonging to each MoG component, given that these spikes belong to the corresponding KS unit. The mean amplitude of each KS unit is shown at the bottom.
